# Epidemiology of fall and its socioeconomic risk factors in community-dwelling Korean elderly

**DOI:** 10.1371/journal.pone.0234787

**Published:** 2020-06-19

**Authors:** Taekyoung Kim, Sang D. Choi, Shuping Xiong

**Affiliations:** 1 Department of Industrial and Systems Engineering, Korea Advanced Institute of Science and Technology (KAIST), Yuseong-gu, Daejoen, Republic of Korea; 2 Department of Occupational and Environmental Safety and Health, University of Wisconsin, Whitewater, Wisconsin, United States of America; Gachon University Gil Medical Center, REPUBLIC OF KOREA

## Abstract

Although falls in older people are a major public health problem globally, to date there are scarce reports on socioeconomic risk factors for falls. The aim of the present study was to investigate the epidemiology of fall, its associated socioeconomic risk factors and relative importance among community-dwelling Korean elderly. Secondary analysis of national survey data with 31,684 community-dwelling Korean elderly was performed. Eleven socioeconomic factors (age, gender, household type, marital status, education level, current occupation, past occupation, income, wealth, number of children, and relationship satisfaction) were selected for analysing their associations with the epidemiology of fall through complex sample analysis and logistic regressions. Results showed that 15.9%~25.1% of community-dwelling Korean elderly experienced fall yearly. The groups with significantly higher fall risks were identified as older aged, being female, not married or widowed, less educated, unemployed, and having lower relationship satisfaction. Gender (adjusted odds ratio-AOR = 1.548) and relationship satisfaction (AOR = 1.276) were the utmost important fall risk factors, indicating being older female with lower relationship satisfaction were the foremost socioeconomic characteristics for risk of falling. These findings could contribute to better understanding of the socioeconomic fall risk profiles among Korean elderly and effective strategies for fall prevention.

## Introduction

Falls among the elderly are a major public health problem. Around one third of older adults aged 65 or over experience at least one fall each year [1,2]. Falls have been reported as a leading cause of fatal death and nonfatal injury in older people [3–5]. Several studies have investigated fall risk factors in community-dwelling older people. Age, gender, historical falls, physical or cognitive impairments, medication, and environmental hazards have been reported as significant risk factors for falls [2,6–10]. Those fall risk factors were widely utilized for not only fall risk assessment, but also management of the risks of falling in the older people [11]. Socioeconomic status of the elderly is the social standing of an individual, which is typically measured by several indicators such as education, occupation, income and wealth. Higher socioeconomic status tends to be positively associated with better well-being and lower nutritional risk [12–15], therefore, older individuals with lower socioeconomic status may have higher fall risks. Nevertheless, very few studies have investigated the association between the elderly’s fall risk and socioeconomic factors, especially for Korean population.

South Korea is facing a rapid population aging [16] and a few studies on elderly fall risk factors applied cross-sectional designs that provide a snapshot of the fall prevalence and risk factors in a single moment of time [17–21]. For example, we conducted a preliminary cross-sectional study at a single moment of time based on 2014 Korean national survey data with 10,451 older individuals [21]. Our earlier results showed that nine out of ten socioeconomic factors were significant for the risk of falls. Although these studies provide valuable insights on association between the risk factors and fall incidents, they typically suffer from selection bias, confounding and cannot identify periodic changes. To overcome the limitations of a single moment cross-sectional study, the use of research designs that provide data at several time points from independent samples of the population (hereafter referred to as “multi-period cross-sectional study”) is increasingly being advocated in the literature [22,23]. A multi-period cross-sectional study can investigate not only association robustly, but also periodic effects.

To date, few studies have been conducted with multi-period cross-sectional designs because the practical application of these multi-period designs is generally considered more complex, expensive, and time consuming. The objective of this study is to investigate the epidemiology of fall, its associated socioeconomic risk factors and relative importance among community-dwelling elderly in South Korea, utilizing a large national database from a multi-period cross-sectional research study.

## Materials and methods

### Description of the data sources

This secondary data analysis used original datasets from 2011 to 2017 *Korean National Survey on Elderly Living Conditions and Welfare Desire (KNSELCWD)* conducted by *Korea Institute for Health and Social Affairs* (KIHASA). KIHASA is a government institute which studies and evaluates national policies and programs related to health care, social welfare, social insurance and population [24]. The original survey data is publicly available through https://data.kihasa.re.kr/index.jsp so that anyone can access, use or share. The surveys were carried out every 3 years, 2011, 2014, and 2017 respectively, to examine conditions about living, relationships, support, health and activities in community-dwelling Korean older population aged 65 or over. The total sample size during the whole study period was 31,684 elderly individuals: 10,997 for year 2011, 10,451 for year 2014, and 10,236 for year 2017. *KNSELCWD* collected fall incidents in the recent one year from each participant.

### Sampling

The target population of the investigation by KIHASA was community-dwelling Korean elderly (aged 65 or over) living in 16 metropolitan cities and provinces. The sampled population was elderly in target population who are residing within areas for sample survey of ‘Population and Housing Census’. Stratified two-stage cluster sampling was used in collecting data. The primary sampling units were 90% of enumeration districts used in ‘Population and Housing Census’ conducted by Statistics Korea and the secondary sampling units were households in the enumeration districts.

There were three steps in computing the required sample size for the *KNSELCWD* in each investigation period. Firstly, total sufficient sample size was computed using Eq (1). With 95% of confidence level and 4% of maximum margin, the sufficient sample size in each region was 600, so total sufficient sample size for 16 stratified regions was 9,600. Afterwards, the total sufficient sample size was allocated to each region in proportion to square root of the actual elderly population size in each region. Lastly, it was ascertained whether the sample size in each region meets the minimum sample size of 384, which was computed again using Eq (1) with 95% of confidence level and 5% of maximum margin. If the sample size in any region was less than 384, extra elderly individuals in the region were added randomly to meet the minimum sample size.

$$N=\frac{{Z_{\alpha /2}}^{2}p(1-p)}{e^{2}}$$ (1)

(Z_α/2_ was a confidence coefficient in a confidence level of (1-α) ×100% 100%, e was the maximum margin of error, and p was population ratio. Because p was an unknown value, this survey assumed p = 0.5 to obtain maximum value of p(1-p)).

### Selection of socioeconomic factors

Studies of socioeconomic inequalities in older people should focus on a set of measures rather than a single indicator [15,25,26]. Education level, occupation (current or past), income, and wealth were the most widely used indicators in assessing individual’s socioeconomic status [27–29]. In addition, important socioeconomic factors including age, gender, type of household, marital status, number of children, and relationship satisfaction with the children were also investigated due to their potential associations with the risk of falling [21,30,31]. Therefore, total 11 socioeconomic factors were chosen in this study to investigate their associations with the fall risk.

### Statistical analysis

**Fig 1:**
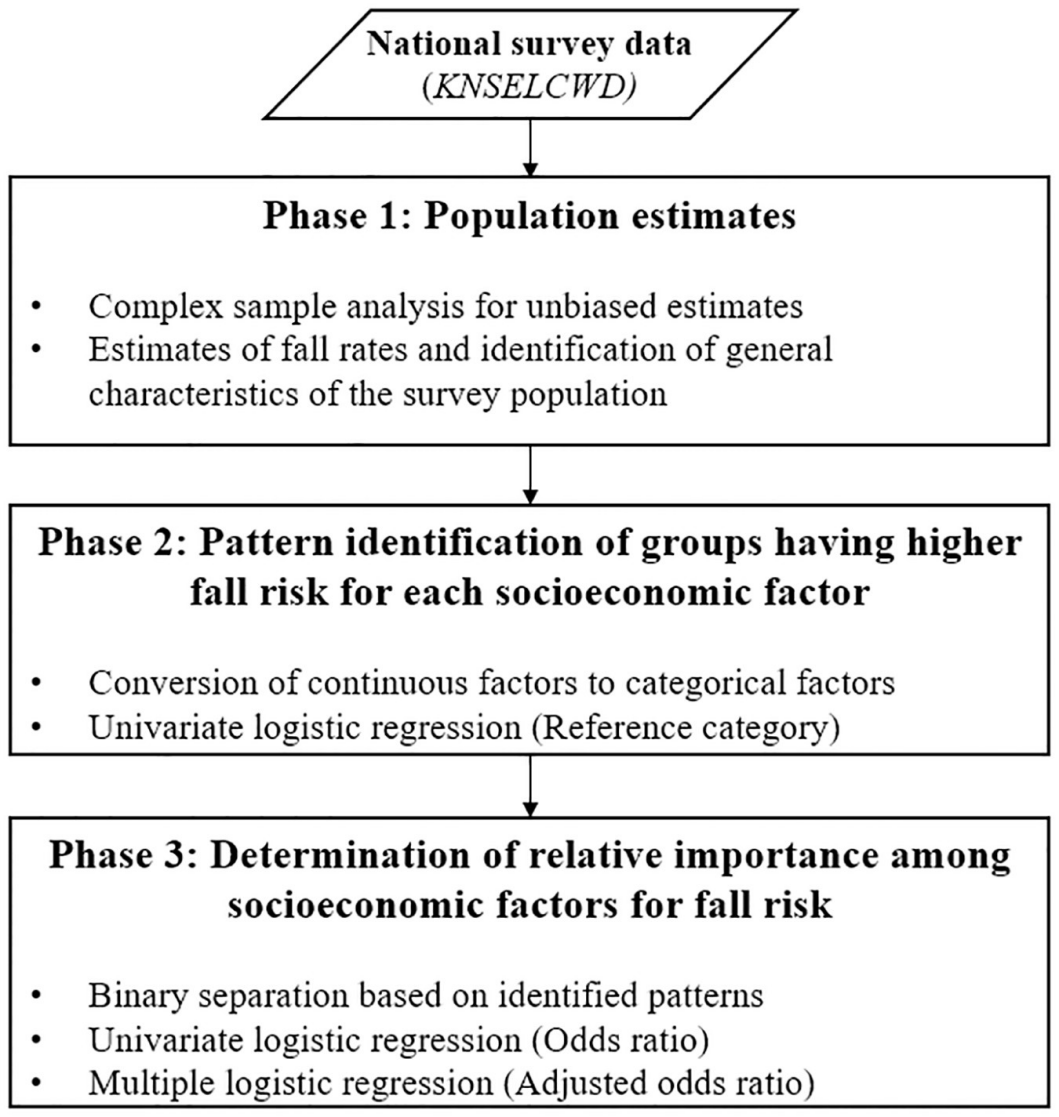
Flow of the overall data analysis process.

Statistical analysis was conducted in three sequential phases as outlined in Fig 1. The first phase was to estimate fall rates and identify general characteristics of the survey population using the complex sample analysis to produce unbiased results [32–34]. The population in each survey year was estimated with weighting and stratifying adjustments. Weights were computed using Eq (2), and stratification was 25 geographic areas consisting of 7 metropolitan cities, and 1 rural area and 1 urban area from each of 9 provinces. In the second phase, patterns about which groups have higher fall risk in each socioeconomic factor were examined using the univariate logistic regression. The continuous factors were converted to categorical factors following the defined categories in *KNSELCWD*. In the univariate logistic regression, one category was set as a reference, and regression coefficients provided the association with the reference category [35]. The odds ratio (OR) greater than 1 indicated that the categories have higher fall risk than the reference category, while OR less than 1 indicated lower fall risk. The last phase was to find out the relative importance among socioeconomic factors using OR and adjusted odds ratio (AOR) of falls. In this phase, all socioeconomic factors were binarily separated into two levels [36], in order to calculate OR and AOR from univariate logistic regression and multiple logistic regression, respectively. Multiple logistic regression was conducted in this study to produce AOR for all socioeconomic factors, which controls the confounding factors so that unbiased relative importance in each factor can be investigated [37]. IBM SPSS Statistics 20 (IBM Corporation, New York, United States) was used for statistical analysis at a significance level of 0.05.

$$Weight=(\frac{S_{h}}{n_{h}S_{hi}}\times \frac{M_{hi}}{m_{hi}})\times \frac{A_{hij}}{a_{hij}}\times w_{ps}$$ (2)

(S_h_: total number of households in strata h, n_h_: the number of enumeration districts in the strata h, S_hi_: the total number of households in the ith enumeration district of the strata h, M_hi_: the number of households in the ith enumeration district of the strata h, m_hi_: the number of investigated households in the ith enumeration district of the strata h, A_hij_: the number of the elderly in the jth household in the ith enumeration district of the strata h, a_hij_: the number of the investigated elderly in the jth household in the ith enumeration district of the strata h, *w*_*ps*_: the post-stratification weight, adjusted by Raking ratio method).

## Results

### Epidemiology of fall and general characteristics of the survey population

Table 1 presents the fall rates and general characteristics of the survey population, segmented by survey year. The estimated fall rates in community-dwelling Korean elderly were 21.2% (95% CI: 20.1–22.2%), 25.1% (95% CI: 24.1–26.1%), and 15.9% (95% CI: 15.1–16.7%) in 2011, 2014, and 2017, respectively. The mean age of Korean elderly was 73.9 years (95% CI: 73.8–74.0) in the whole period, 57.5% were female (95% CI: 56.9–58.2%).

**Table 1:** Fall rates and general characteristics of the survey population, segmented by survey year.

Years	2011 (95% CI)	2014 (95% CI)	2017 (95% CI)	The whole period (95% CI)
Characteristics				
Estimate of Korean older population	7,070,586	6,385,559	7,075,518	20,531,663
	(6,974,827–7,166,345)	(6,323,461–6,447,657)	(7,047,651–7,103,384)	(20,409,302–20,654,024)
Fall rate (%)	21.2 (20.1–22.2)	25.1 (24.1–26.1)	15.9 (15.1–16.7)	20.6 (20.0–21.1)
Mean age (years)	73.7 (73.5–73.8)	73.9 (73.7–74.0)	74.1 (73.9–74.2)	73.9 (73.8–74.0)
Gender				
Male (%)	43.1 (41.9–44.4)	41.7 (40.5–42.8)	42.5 (41.4–43.6)	42.5 (41.8–43.1)
Female (%)	56.9 (55.6–58.1)	58.3 (57.2–59.5)	57.5 (56.4–58.6)	57.5 (56.9–58.2)
Household type				
Living alone (%)	19.6 (18.7–20.5)	23.0 (22.1–24.0)	23.6 (22.7–24.5)	22.0 (21.5–22.6)
Living with the spouse (%)	48.5 (47.2–49.7)	44.5 (43.4–45.7)	48.4 (47.3–49.4)	47.2 (46.5–47.9)
Living with the children (%)	27.3 (26.2–28.5)	28.4 (27.4–29.5)	23.7 (22.8–24.7)	26.4 (25.8–27.0)
Living with others (%)	4.6 (4.1–5.2)	4.0 (3.5–4.5)	4.4 (3.9–4.8)	4.3 (4.0–4.6)
Marital status				
Not married (%)	0.3 (0.2–0.5)	0.4 (0.3–0.6)	0.5 (0.4–0.7)	0.4 (0.3–0.5)
Married (%)	67.5 (66.4–68.6)	61.4 (60.3–62.6)	63.4 (62.3–64.4)	64.2 (63.6–64.8)
Widowed (%)	30.3 (29.2–31.4)	34.2 (33.1–35.3)	31.5 (30.5–32.5)	31.9 (31.3–32.6)
Divorced (%)	1.3 (1.1–1.6)	3.0 (2.6–3.4)	3.6 (3.2–4.0)	2.6 (2.4–2.8)
Living separately (%)	0.5 (0.4–0.8)	1.0 (0.8–1.2)	1.0 (0.8–1.3)	0.8 (0.7–1.0)
Educational level				
Uneducated (illiterate) (%)	11.0 (10.3–11.6)	9.6 (9.0–10.3)	6.6 (6.1–7.1)	9.0 (8.7–9.4)
Uneducated (literate) (%)	20.7 (19.8–21.7)	20.9 (20.0–21.8)	17.6 (16.9–18.4)	19.7 (19.2–20.2)
Elementary school (%)	35.4 (34.2–36.7)	32.0 (31.0–33.1)	34.1 (33.1–35.1)	33.9 (33.3–34.6)
Middle school (%)	13.4 (12.5–14.4)	13.2 (12.4–14.0)	16.8 (16.0–17.7)	14.5 (14.0–15.0)
High school (%)	12.5 (11.6–13.5)	16.6 (15.7–17.5)	17.3 (16.5–18.1)	15.4 (14.9–16.0)
College (%)	1.2 (0.9–1.5)	1.2 (0.9–1.4)	1.0 (0.8–1.2)	1.1 (1.0–1.3)
University or higher (%)	5.8 (5.1–6.5)	6.6 (6.0–7.2)	6.5 (6.0–7.1)	6.3 (5.9–6.7)
Current employment status				
Employed (%)	33.6 (32.4–34.8)	28.5 (27.5–29.5)	30.3 (29.3–31.3)	30.9 (30.3–31.5)
Unemployed (%)	66.4 (65.2–67.6)	71.5 (70.5–72.5)	69.7 (68.7–70.7)	69.1 (68.5–69.7)
Past career				
Inoccupation (%)	9.5 (8.7–10.2)	10.8 (10.1–11.6)	10.5 (9.9–11.2)	10.2 (9.8–10.7)
Managers (%)	3.0 (2.6–3.6)	4.2 (3.7–4.8)	2.6 (2.3–3.0)	3.3 (3.0–3.5)
Professionals (%)	3.4 (2.9–3.9)	5.4 (4.9–6.0)	4.6 (4.2–5.1)	4.4 (4.2–4.7)
Clerks (%)	4.4 (3.8–5.0)	4.3 (3.8–4.8)	5.9 (5.4–6.4)	4.9 (4.6–5.2)
Service workers (%)	5.6 (5.0–6.2)	6.9 (6.3–7.5)	7.7 (7.1–8.3)	6.7 (6.4–7.1)
Sales workers (%)	10.8 (10.0–11.6)	11.2 (10.5–12.0)	10.7 (10.1–11.4)	10.9 (10.5–11.4)
Skilled agricultural, forestry and fishery workers (%)	34.5 (33.6–35.5)	25.0 (24.1–25.8)	22.7 (22.0–23.4)	27.5 (27.0–28.0)
Craft and related trades workers (%)	8.6 (7.8–9.4)	8.6 (8.0–9.3)	9.0 (8.4–9.7)	8.7 (8.3–9.2)
Equipment, machine operating and assessing workers (%)	3.9 (3.3–4.6)	6.0 (5.5–6.6)	6.8 (6.3–7.4)	5.5 (5.2–5.9)
Elementary workers (%)	15.7 (14.8–16.6)	17.0 (16.2–17.9)	18.8 (18.0–19.6)	17.2 (16.7–17.7)
Armed Forces (%)	0.7 (0.5–0.9)	0.6 (0.4–0.8)	0.6 (0.5–0.8)	0.6 (0.5–0.8)
Annual income (10,000 Korean won)^a^	849.8 (821.8–877.8)	959.3 (924.8–993.8)	1176.5 (1143.6–1209.4)	996.4 (978.0–1014.8)
Personal wealth (10,000 Korean won)^a^	15180.1 (14181.2–16179.1)	13857.8 (13056.7–14658.9)	28144.5 (27062.6–29226.4)	19246.6 (18674.4–19818.9)
Number of children	3.7 (3.7–3.8)	3.4 (3.4–3.4)	3.2 (3.2–3.3)	3.5 (3.4–3.5)
Relationship satisfaction				
Very satisfied (%)	4.3 (3.8–4.9)	2.0 (1.7–2.4)	1.6 (1.4–2.0)	2.7 (2.4–2.9)
Satisfied (%)	56.9 (55.6–58.1)	40.7 (39.5–41.8)	47.0 (45.9–48.0)	48.4 (47.7–49.1)
Moderate (%)	31.8 (30.6–33.0)	47.0 (45.8–48.2)	42.8 (41.8–43.9)	40.4 (39.7–41.0)
Rarely satisfied (%)	6.2 (5.7–6.9)	9.5 (8.8–10.3)	8.0 (7.4–8.6)	7.9 (7.5–8.2)
Not satisfied (%)	0.8 (0.6–1.0)	0.8 (0.6–1.0)	0.6 (0.5–0.8)	0.7 (0.6–0.8)

^a^ 1USD ≈ 1200 Korean won

### Major socioeconomic factors related to fall risk in community-dwelling Korean elderly

#### Patterns of socioeconomic factors associated with higher fall risk

The univariate logistic regression was used to discover patterns of higher fall risk groups by each socioeconomic factor (Table 2). Groups with higher risk of falling were identified as older aged, being female, living alone, not married or widowed, less educated, unemployed, having had blue-collar jobs, with lower income and wealth, having lower relationship satisfaction, and having more children.

**Table 2:** Results of the univariate logistic regression for identifying higher fall risk groups associated with the 11 selected socioeconomic factors (OR > 1 indicates higher fall risk than the reference category while OR < 1 indicates lower fall risk).

Years	2011		2014		2017		The whole period	
Factors	OR (95%CI)	p value	OR (95%CI)	p value	OR (95%CI)	p value	OR (95%CI)	p value
Age (reference: 65–69 years old)								
70–74 years old	1.238 (1.036–1.479)	0.019	1.332 (1.147–1.548)	0.000	1.271 (1.069–1.511)	0.007	1.295 (1.176–1.425)	0.000
75–79 years old	1.635 (1.366–1.959)	0.000	1.598 (1.372–1.862)	0.000	1.517 (1.281–1.795)	0.000	1.588 (1.441–1.749)	0.000
≥80 years old	1.637 (1.355–1.979)	0.000	1.874 (1.590–2.208)	0.000	1.920 (1.620–2.275)	0.000	1.796 (1.624–1.986)	0.000
Gender (reference: male)								
Female	2.020 (1.760–2.317)	0.000	2.075 (1.847–2.331)	0.000	1.898 (1.671–2.156)	0.000	1.998 (1.856–2.151)	0.000
Household type (reference: living alone)								
Living with the spouse	0.598 (0.517–0.692)	0.000	0.585 (0.512–0.668)	0.000	0.585 (0.509–0.672)	0.000	0.591 (0.546–0.641)	0.000
Living with the children	0.802 (0.683–0.943)	0.007	0.833 (0.720–0.965)	0.015	0.798 (0.680–0.936)	0.006	0.832 (0.761–0.910)	0.000
Living with others	0.654 (0.480–0.891)	0.007	0.554 (0.404–0.759)	0.000	0.642 (0.458–0.901)	0.010	0.626 (0.520–0.753)	0.000
Marital status (reference: married)								
Not married	3.044 (1.283–7.222)	0.012	1.778 (0.863–3.663)	0.118	1.751 (0.793–3.867)	0.166	1.968 (1.257–3.080)	0.003
Widowed	1.637 (1.447–1.852)	0.000	1.900 (1.697–2.128)	0.000	1.958 (1.735–2.210)	0.000	1.825 (1.703–1.955)	0.000
Divorced	1.133 (0.697–1.841)	0.614	1.281 (0.930–1.764)	0.129	1.518 (1.095–2.104)	0.012	1.280 (1.041–1.572)	0.019
Living separately	1.255 (0.551–2.856)	0.589	1.597 (0.936–2.726)	0.086	0.566 (0.273–1.175)	0.127	1.113 (0.764–1.621)	0.579
Education level (reference: university or higher)								
Uneducated (illiterate)	2.516 (1.643–3.853)	0.000	3.270 (2.383–4.486)	0.000	2.102 (1.487–2.971)	0.000	2.723 (2.211–3.354)	0.000
Uneducated (literate)	2.257 (1.482–3.438)	0.000	2.667 (1.980–3.590)	0.000	2.070 (1.514–2.830)	0.000	2.365 (1.936–2.887)	0.000
Elementary school	1.690 (1.115–2.563)	0.013	2.029 (1.512–2.722)	0.000	1.553 (1.145–2.106)	0.005	1.748 (1.436–2.128)	0.000
Middle school	1.221 (0.775–1.922)	0.389	1.398 (1.014–1.928)	0.041	1.187 (0.855–1.649)	0.307	1.246 (1.006–1.544)	0.044
High school	1.273 (0.801–2.022)	0.307	1.384 (1.006–1.905)	0.046	1.235 (0.888–1.718)	0.209	1.293 (1.043–1.602)	0.019
College	0.906 (0.413–1.988)	0.805	1.229 (0.611–2.472)	0.563	0.919 (0.394–2.141)	0.845	1.032 (0.663–1.607)	0.889
Current employment status (reference: employed)								
Unemployed	1.575 (1.378–1.799)	0.000	1.507 (1.334–1.703)	0.000	1.432 (1.252–1.636)	0.000	1.514 (1.405–1.632)	0.000
Past career (reference: managers)								
Inoccupation	2.362 (1.361–4.101)	0.002	2.252 (1.517–3.345)	0.000	1.384 (0.901–2.124)	0.137	1.949 (1.495–2.540)	0.000
Professionals	1.344 (0.685–2.637)	0.390	1.161 (0.742–1.817)	0.514	0.980 (0.594–1.616)	0.937	1.135 (0.833–1.547)	0.421
Clerks	1.164 (0.601–2.253)	0.653	0.956 (0.597–1.531)	0.853	1.086 (0.677–1.742)	0.733	1.034 (0.759–1.409)	0.831
Service workers	2.055 (1.158–3.646)	0.014	2.156 (1.435–3.238)	0.000	1.386 (0.892–2.155)	0.147	1.782 (1.356–2.341)	0.000
Sales workers	1.772 (1.021–3.073)	0.042	1.970 (1.328–2.921)	0.001	1.580 (1.032–2.419)	0.035	1.740 (1.336–2.265)	0.000
Skilled agricultural, forestry and fishery workers	1.981 (1.173–3.347)	0.011	2.132 (1.472–3.088)	0.000	1.438 (0.961–2.150)	0.077	1.820 (1.418–2.337)	0.000
Craft and related trades workers	1.391 (0.782–2.472)	0.261	1.248 (0.824–1.892)	0.296	0.880 (0.557–1.390)	0.583	1.139 (0.861–1.506)	0.361
Equipment, machine operation and assembling workers	1.307 (0.629–2.715)	0.474	1.310 (0.843–2.037)	0.230	0.940 (0.589–1.501)	0.797	1.138 (0.836–1.549)	0.412
Elementary workers	1.959 (1.144–3.356)	0.014	2.205 (1.507–3.228)	0.000	1.564 (1.039–2.354)	0.032	1.845 (1.428–2.383)	0.000
Armed Forces	0.914 (0.345–2.425)	0.857	0.898 (0.349–2.306)	0.822	1.156 (0.481–2.776)	0.746	0.959 (0.561–1.640)	0.877
Annual income^#^ (reference: high income)								
Middle income	1.792 (1.503–2.135)	0.000	1.892 (1.631–2.194)	0.000	1.585 (1.354–1.855)	0.000	1.758 (1.601–1.929)	0.000
Low income	1.838 (1.509–2.237)	0.000	1.990 (1.685–2.350)	0.000	1.483 (1.238–1.777)	0.000	1.774 (1.597–1.970)	0.000
Personal wealth^#^ (reference: high wealth)								
Middle wealth	1.420 (1.191–1.694)	0.000	1.410 (1.221–1.628)	0.000	1.196 (1.019–1.404)	0.028	1.340 (1.222–1.470)	0.000
Low wealth	1.999 (1.652–2.421)	0.000	1.743 (1.490–2.039)	0.000	1.451 (1.218–1.728)	0.000	1.733 (1.568–1.917)	0.000
Number of children^#^ (reference: small size)								
Middle size	1.089 (0.952–1.246)	0.214	1.163 (1.017–1.330)	0.027	0.953 (0.829–1.095)	0.496	1.073 (0.992–1.161)	0.079
Large size	1.360 (1.142–1.620)	0.001	1.305 (1.119–1.522)	0.001	1.377 (1.167–1.626)	0.000	1.364 (1.242–1.499)	0.000
Relationship satisfaction^§^ (reference: satisfied)								
Moderate	1.197 (1.045–1.372)	0.010	1.327 (1.180–1.439)	0.000	1.298 (1.145–1.472)	0.000	1.272 (1.184–1.367)	0.000
Not satisfied	1.566 (1.248–1.964)	0.000	2.159 (1.796–2.596)	0.000	2.019 (1.653–2.466)	0.000	1.931 (1.721–2.167)	0.000

^#^Continuous factors were categorized into 3 groups based on quartiles (Low/small: less than the first quartile Q1; High/large: larger than the third quartile Q3; Middle: in between Q1 and Q3).

^§^Due to too limited samples in two extreme categories (very satisfied and not satisfied) in relationship satisfaction, original five categories were regrouped into three categories to increase the sample size in each category for robust comparisons. ‘very satisfied’ and ‘satisfied’ were combined to a new category of ‘satisfied’, ‘rarely satisfied’ and ‘not satisfied’ were combined to ‘not satisfied’, while the original category of ‘moderate’ was kept unchanged.

#### Relative importance of socioeconomic factors for elderly fall risks

Table 3 summarizes the criteria used to generate binary groups based on each socioeconomic factor and Table 4 further shows the odds ratio (OR) and adjusted odds ratio (AOR) of falls for each socioeconomic factor based on the binary separation. All 11 socioeconomic factors showed statistical significance for fall risks from the univariate logistic regression analysis in the whole period, especially for gender (OR = 2.004), marital status (OR = 1.766), education level (OR = 1.675) and past career (OR = 1.614). However, after adjusting for potentially confounding effects from other socioeconomic factors by multiple logistic regression, 5 factors (‘household type’, ‘past career’, ‘income’, ‘wealth’, and ‘number of children’) became statistically insignificant for fall risks. ‘Gender’ still recorded the highest importance (AOR = 1.548) in the whole period, followed by ‘relationship satisfaction’ (AOR = 1.276), ‘marital status’ (AOR = 1.251), ‘age’ (AOR = 1.245), ‘current employment status’ (AOR = 1.236) and ‘education level’ (AOR = 1.206).

**Table 3:** Binary separation of the socioeconomic factors to examine relative importance for fall risks.

Socioeconomic factors		Binary separation
Categorical type *(7 factors)*	Gender	Male / female
	Household type	Living with someone / living alone
	Marital status	Married / not married or with change in marriage
	Education level^#^	Higher / lower than the middle school level
	Current employment status	Employed / unemployed
	Past career	White-collar / blue-collar or jobless
	Relationship satisfaction	Satisfied / from not satisfied to moderately satisfied
Continuous type *(4 factors)*	Age (years)	Younger /older than median age (73 in 2011; 73 in 2014; 73 in 2017)
	Annual income (10,000 Korean won)	Higher / lower than median income (548 in 2011; 590 in 2014; 766 in 2017)
	Personal wealth (10,000 Korean won)	Higher / lower than median wealth (3,000 in 2011; 3,130 in 2014; 14,200 in 2017)
	Number of children	Smaller / larger than median number of children (4 in 2011; 3 in 2014; 3 in 2017)

^#^Compulsory education in South Korea is until year 9 (middle school).

**Table 4:** Relative importance of socioeconomic factors for elderly fall risks.

Years	2011		2014		2017		The whole period	
Factors	OR	AOR	OR	AOR	OR	AOR	OR	AOR
Age	1.470*	1.266*	1.476*	1.143*	1.574*	1.340*	1.501*	1.245*
	(1.299–1.663)	(1.103–1.453)	(1.325–1.645)	(1.012–1.290)	(1.397–1.775)	(1.171–1.533)	(1.402–1.606)	(1.154–1.343)
Gender	2.020*	1.644*	2.075*	1.449*	1.898*	1.542*	2.004*	1.548*
	(1.760–2.317)	(1.380–1.958)	(1.847–2.331)	(1.256–1.671)	(1.671–2.156)	(1.324–1.796)	(1.861–2.158)	(1.416–1.692)
Household type	1.497*	1.092	1.492*	1.030	1.533*	0.994	1.506*	1.032
	(1.315–1.704)	(0.895–1.332)	(1.322–1.684)	(0.868–1.223)	(1.351–1.740)	(0.821–1.202)	(1.401–1.619)	(0.927–1.149)
Marital status	1.619*	1.038	1.838*	1.345*	1.864*	1.426*	1.766*	1.251*
	(1.434–1.828)	(0.857–1.258)	(1.647–2.051)	(1.141–1.585)	(1.657–2.098)	(1.185–1.717)	(1.651–1.889)	(1.128–1.388)
Education level	1.671*	1.257*	1.834*	1.240*	1.506*	1.122	1.675*	1.206*
	(1.429–1.953)	(1.046–1.510)	(1.624–2.070)	(1.068–1.439)	(1.329–1.708)	(0.956–1.317)	(1.549–1.811)	(1.097–1.325)
Current employment status	1.575*	1.348*	1.507*	1.198*	1.432*	1.151	1.510*	1.236*
	(1.378–1.799)	(1.168–1.556)	(1.334–1.703)	(1.051–1.366)	(1.252–1.636)	(0.998–1.328)	(1.401–1.628)	(1.140–1.340)
Past career	1.616*	1.088	1.880*	1.174	1.325*	0.959	1.614*	1.073
	(1.238–2.109)	(0.800–1.481)	(1.561–2.265)	(0.946–1.457)	(1.093–1.605)	(0.769–1.196)	(1.425–1.827)	(0.931–1.238)
Annual income	1.406*	0.982	1.534*	1.191*	1.293*	1.020	1.417*	1.066
	(1.242–1.592)	(0.853–1.131)	(1.376–1.710)	(1.050–1.351)	(1.150–1.454)	(0.890–1.168)	(1.324–1.516)	(0.986–1.152)
Personal wealth	1.508*	1.079	1.526*	1.125	1.222*	0.962	1.426*	1.070
	(1.331–1.709)	(0.933–1.247)	(1.368–1.702)	(0.994–1.274)	(1.086–1.375)	(0.841–1.100)	(1.332–1.527)	(0.992–1.153)
Number of children	1.219*	1.047	1.206*	0.991	1.239*	0.997	1.220*	1.007
	(1.075–1.383)	(0.913–1.202)	(1.083–1.343)	(0.881–1.114)	(1.100–1.395)	(0.874–1.138)	(1.140–1.305)	(0.935–1.085)
Relationship satisfaction	1.260*	1.196*	1.459*	1.302*	1.410*	1.346*	1.371*	1.276*
	(1.111–1.430)	(1.050–1.363)	(1.303–1.634)	(1.159–1.463)	(1.251–1.589)	(1.184–1.529)	(1.280–1.469)	(1.188–1.371)

* p<0.05;

AOR of each investigated factor was the odds ratio adjusted by all other factors (confounders) and survey year.

## Discussion

The estimated fall rate increased by 3.9% from 2011 to 2014, but subsequently drastically dropped by 9.2% from 2014 to 2017. It seemed worthy of a further investigation to discover potential reasons for this sudden drop of fall rate in 2017. To begin with, we examined the participants’ physical conditions including subjective health condition from *KNSELCWD* (2011, 2014, & 2017) and determined that physical functionality and subjective health condition did not have any significant differences over the study period. Yet, *KNSELCWD* examined the impaired level of cognitive functionality of the Korean elderly people using a Korean version of mini mental state examination. Results showed that the estimated proportions of the elderly having low cognitive function were 20.5% (95% CI: 19.5–21.6%), 25.1% (95% CI: 24.2–26.1%) in 2011, 2014, and unexpectedly dropped to 15.1% (95% CI: 14.3–15.8%) in 2017, which closely follows a similar trend to that of the fall rates. Previous literature documented that low cognitive function is one of the major fall risk factors in the elderly people since cognitive impairment can delay the sensory integration and the selection/execution of proper corrective responses to prevent falls, especially for the critical situations such as slips, trips and missteps [17,38–40]. Moreover, as a response to rapidly growing the Korean elderly population, in 2015 Korean government announced key policies for the revitalization of the health welfare associated with the elderly’s loneliness, deterioration in health, poverty, depression, dementia, and cognitive impairment [41]. We suspect that Korean government’s policies for the revitalization of the elderly’s health welfare initiatives might contribute to the reduction of proportion of elderly with cognitive impairment, and thus concurrently affected to the substantial decrease of fall rates in 2017 [16].

The identified higher fall risk groups in regard to socioeconomic factors included older aged, being female, living alone, not married, less educated, unemployed, having had blue-collar career, with lower income and wealth, with lower relationship satisfaction, and having more children. This is consistent with the findings from our earlier single-moment cross-sectional study [21] except on the number of children (see Table 2). Previous literature reported that aging is a widely known fall risk factor [42–44] and older women are more prone to falling and injury than men [42,44–46]. This study also found that socioeconomic conditions of less education and lower income/wealth were associated with higher risk of falling. Low education level has been reported as an important indicator of cognitive impairment in older people [47] and highly educated elderly are more aware of the risk associated with falling and they can access education or other strategies to protect against fall risk [48]. In addition, older people with low income and wealth were at high risk of falling. This result is expected since the elderly in poverty are exposed to more environmental hazards and have lower accessibility to health care services, which induce greater risk of chronical diseases, limitation on physical condition and increased fall risks [3,29,46]. Importantly, our study found that ‘marital status’ and ‘relationship satisfaction with the children’ were the main socioeconomic factors for the risk of falling. Those two factors are associated with social support from the family and life satisfaction, older people having good relationship with the family and high life satisfaction tend to suffer less from isolation, depression and risk of falling [3,49]. Earlier studies also showed that ‘marital status’ was associated with falls [44,48] and widowed/separated/divorced people did not have benefits about healthy behaviors such as good diet and physical activity [42]. Moreover, employed older people tend to have lower fall risks than unemployed older people. Generally, elderly who employed are considered to have sufficient capability to perform daily activities, and they have higher life satisfaction [50]. In addition, employed older people have more opportunities to interact with other people [51], the social interactions can help avoid social isolation and depression, thus reduce the risk of falling.

After identifying significant socioeconomic factors for elderly fall risks [21], it is important to further check their relative importance so that the measures/policies for fall prevention can be prioritized accordingly. In the present study, there are considerable differences between results of OR and AOR for relative importance of socioeconomic factors (Table 4). In the whole period, all the socioeconomic factors were significant in terms of OR, but AOR of ‘household type’, ‘past career’, ‘number of children’ ‘income’, and ‘wealth’ became insignificant after removing confounders. To gain insights for this phenomenon, multivariate logistic regression analyses were conducted to check the confounding effects among socioeconomic factors. Results showed that ‘past career’ was highly affected by education level and gender. In general, highly educated people had white-collar career while lowly educated people had blue-collar career in past Korea. Also males had higher chance to get jobs while females concentrated on housework. Household type was dominantly associated with marital status. Married people typically lived with their spouse, and unmarried/divorced/widowed people generally lived alone. The number of children was influenced by age and education level. Traditionally, Korean formed large family because they need hands for helping the manual labour such as farming, but the trend changed due to the rapid industrialization and economic take off in Korea, which began in the early 1960s. In addition, gender and current employment status were identified to have a dominant influence on income and wealth. Therefore, the significance of ‘past career’, ‘household type’, ‘number of children’, ‘income’ and ‘wealth’ on elderly fall risks (indicated by univariate logistic regression) was likely due to the confounding effects from other relevant socioeconomic factors (education level, gender, marital status, age, current employment status etc.).

After adjusting for potentially confounding effects, gender was found to be the most important socioeconomic factor related to fall risk in Korean elderly (AOR in the whole periods = 1.548), followed by relationship satisfaction (AOR = 1.276), marital status (AOR = 1.251), age (AOR = 1.245), and current employment status (AOR = 1.236), with education level (AOR = 1.206) being statistically significant but least important. Therefore, it is possible to infer being older female with lower relationship satisfaction were the foremost socioeconomic characteristics for risk of falling in community-dwelling Korean elderly. Surprisingly, even though age has been considered as an important risk factor in elderly’s falls by many earlier studies, its effect after the adjustment, though still statistically significant, was found to be only moderate in this study (AOR = 1.245). This result is consistent with the findings from Oh et al. [49] and Bueno-Cavanillas et al. [52]. We believe the fundamental reason that age is a major fall risk factor in older people is the decline in physical, sensory and cognitive functions due to aging rather than age itself.

Some limitations are inherent within this study. Firstly, the selected factors for socioeconomic status are not fully representative. Some important factors such as home environment and religion were not covered in this study due to the lack of corresponding data. Secondly, due to the nature of retrospective study, we can only determine the associations between the socioeconomic factors and the risk of falling for the Korean elderly, not the causation. Last but not least, caution should be taken in generalizing the findings from this study to elderly populations in other countries because socioeconomic characteristics can vary from different countries or cultures.

## Conclusions

The estimated annual fall rates of community-dwelling Korean elderly ranged from 15.9% to 25.1%. The groups with higher fall risks were identified as older aged, being female, not married or widowed, less educated, unemployed, and having lower relationship satisfaction. Gender (AOR = 1.548) and relationship satisfaction (AOR = 1.276) were the utmost important fall risk factors. Therefore, it is possible to infer being older female with lower relationship satisfaction were the foremost socioeconomic characteristics for risk of falling in community-dwelling Korean elderly. These findings could contribute to better understanding of the socioeconomic fall risk profiles among Korean elderly and effective strategies for fall prevention.
